# Associations of birthweight and history of childhood obesity with beta cell mass in Japanese adults

**DOI:** 10.1007/s00125-020-05127-2

**Published:** 2020-04-01

**Authors:** Hironobu Sasaki, Yoshifumi Saisho, Jun Inaishi, Yuusuke Watanabe, Tami Tsuchiya, Masayoshi Makio, Midori Sato, Minoru Kitago, Taketo Yamada, Hiroshi Itoh

**Affiliations:** 1 Department of Internal Medicine, Keio University School of Medicine, 35 Shinanomachi, Shinjuku-ku, Tokyo, 160-8582 Japan; 2 Department of Surgery, Keio University School of Medicine, Tokyo, Japan; 3 Department of Pathology, Keio University School of Medicine, Tokyo, Japan; 4 Department of Pathology, Saitama Medical University, Saitama, Japan

**Keywords:** Alpha cell mass, Beta cell mass, Birthweight, Human pancreas, Islet size, Japanese cohort, Obesity

## Abstract

**Aims/hypothesis:**

Low birthweight is associated with a high risk of diabetes, but there are no reports discussing birthweight and pancreatic tissues in humans. The purpose of this study was to examine the correlation between birthweight and beta and alpha cell mass in humans.

**Methods:**

Sixty-four Japanese adults with and without diabetes who underwent pancreatectomy and were able to recall their weight history including birthweight were included. Pancreatic tissues were stained for insulin and glucagon, and fractional beta cell area (BCA) and alpha cell area (ACA) were quantified. Islet size and density and beta cell replication were also quantified and their associations with birthweight were evaluated.

**Results:**

In participants without diabetes, there was a weak positive correlation between birthweight and BCA (*R* = 0.34, *p* = 0.03). The group with a history of childhood obesity, but not the group with a history of obesity in adulthood only, showed higher BCA compared with those without a history of obesity (1.78 ± 0.74% vs 0.99 ± 0.53%, *p* = 0.01), and the correlation coefficient between birthweight and BCA increased after excluding those with a history of childhood obesity (*R* = 0.51, *p* < 0.01). In those with diabetes, there was no correlation between birthweight and BCA. No correlation was found between birthweight and ACA in either those with or without diabetes.

**Conclusions/interpretation:**

Birthweight and beta, but not alpha, cell mass are positively correlated in non-diabetic adults, and a history of childhood obesity may affect beta cell mass.

**Figure:**
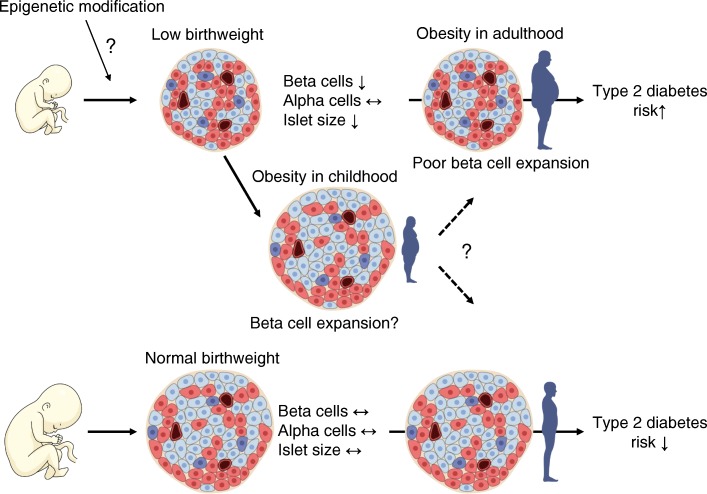
Graphical abstract

**Electronic supplementary material:**

The online version of this article (10.1007/s00125-020-05127-2) contains peer-reviewed but unedited supplementary material, which is available to authorised users.

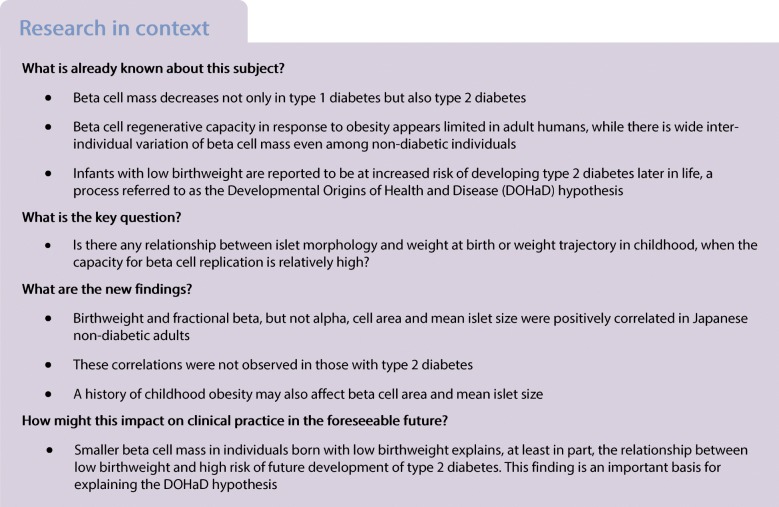



## Introduction

It has been reported that beta cell mass (BCM) decreases not only in type 1 diabetes but also type 2 diabetes [1–4]. Because the prevalence of type 2 diabetes is increasing worldwide, it is important to develop a strategy to preserve and restore BCM.

Normally, the decrease in insulin sensitivity induced by obesity is compensated for by increasing insulin secretion to maintain normal glucose tolerance [5]. However, it has been suggested that, in contrast to rodents, the increase in BCM in the presence of obesity is relatively small in humans. In the Europid population, it has been reported that BCM increases by approximately 20–50% in obese individuals without diabetes [6, 7], while we have previously reported that no significant increase in BCM occurs in obese non-diabetic Japanese adults [3, 8]. We have also shown that in non-diabetic Japanese individuals, corticosteroid administration, which induces insulin resistance, did not increase BCM [9], suggesting that beta cell regenerative capacity is very limited especially in this population. However, these studies have evaluated obesity and/or insulin resistance only in adulthood, when beta cell replication ability has decreased [6, 8, 10, 11]. In view of the wide inter-individual variation of BCM even in the non-diabetic population, exploring the factors that affect BCM will have a significant impact on prevention and treatment strategies in diabetes.

In 1991, Hales et al. showed a negative correlation between birthweight and the onset of type 2 diabetes [12]. Subsequently, the Developmental Origins of Health and Disease (DOHaD) hypothesis has been proposed: that the risk of developing adult-onset non-communicable diseases, such as type 2 diabetes, hypertension, dyslipidaemia, ischaemic heart disease and kidney disease is affected by fetal development [13, 14]. Although the underlying mechanism of the DOHaD hypothesis has not been fully elucidated, it has been recently reported that birthweight correlates with glomerular number in the kidney [15], suggesting that birthweight affects organ development, which leads to susceptibility to the future development of diseases. However, there are no reports examining the relation between birthweight and pancreatic tissues in humans.

Therefore, in this study, to elucidate the mechanism of the association between low birthweight and future risk of type 2 diabetes, we sought to address the following questions by using pancreatic tissues resected by surgery: (1) Is there any correlation between birthweight and BCM? (2) Is there any correlation between birthweight and alpha cell mass (ACM)? (3) Does a history of childhood obesity affect them?

## Methods

### Participants

This study was approved by the Ethics Committee of Keio University School of Medicine. From May 2012 to March 2019, 401 patients underwent pancreatectomy at Keio University Hospital. Of those, we included 64 Japanese patients with and without diabetes who could recall their weight history including birthweight (43 men and 21 women) in this study. The inclusion criteria were: (1) ability to recall their weight history in childhood and birthweight to at least 100 g; (2) gave written informed consent; and (3) the pancreatic tissue contained adequate amounts of normal pancreas for histological analysis. The exclusion criteria were: (1) type 1 diabetes; or (2) a functional neuroendocrine tumour such as insulinoma or glucagonoma. All of the patients with diabetes had been diagnosed with type 2 diabetes before the diagnosis of pancreatic disease (mean duration of diabetes 8.8 ± 5.9 years, Table 1). Some of the participants included in this study (*n* = 11) have also been included in our prior study [3].

**Table 1:** Participant characteristics

Characteristic	NDM group	DM group	Total
*N* (male/female)	38 (20/18)	26 (23/3)	64 (43/21)
Age, years	61.7 ± 14.3	67.2 ± 11.2	63.9 ± 13.3
Current BMI, kg/m^2^	22.3 ± 3.6	25.1 ± 3.5**	23.4 ± 3.8
Maximum BMI, kg/m^2^	24.9 ± 4.0	28.5 ± 3.8**	26.3 ± 4.3
HbA_1c_, mmol/mol	38 ± 4	51 ± 10**	43 ± 10
HbA_1c_, %	5.7 ± 0.5	6.9 ± 1.0**	6.2 ± 0.9
Glycated albumin, % ^a^	15.2 ± 2.3	18.9 ± 3.5*	17.6 ± 3.6
Plasma glucose, mmol/l ^b^	6.0 ± 0.8	7.8 ± 2.3**	6.7 ± 1.8
Clinical diagnosis, *n* (%)			
Pancreatic cancer	13 (34)	14 (54)	27 (42)
IPMN	8 (21)	4 (15)	12 (19)
Non-functional neuroendocrine tumour	9 (24)	2 (8)	11 (17)
Bile duct cancer	3 (8)	2 (8)	5 (8)
Duodenal papilla cancer	1 (3)	1 (4)	2 (3)
Other ^c^	4 (11)	3 (12)	7 (11)
Operative procedure, *n* (%)			
Pancreatoduodenectomy	24 (63)	16 (62)	40 (63)
Distal pancreatectomy	12 (32)	8 (31)	20 (31)
Total pancreatectomy	2 (5)	2 (8)	4 (6)
Birthweight, g	3023 ± 439	3030 ± 511	3026 ± 466
History of obesity, *n* (%)			
Up to adulthood	6 (16)	9 (35)	15 (23)
Early childhood	2 (5)	4 (15)	6 (9)
Elementary school age	4 (11)	4 (15)	8 (13)
Junior high school age	4 (11)	4 (15)	8 (13)
High school age	3 (8)	8 (31)	11 (17)
Adulthood	15 (39)	24 (92)	39 (61)
Obesity history throughout life	18 (47)	24 (92)	42 (66)
Duration of diabetes, years	–	8.8 ± 5.9	–
Family history of diabetes in second-degree relative, *n* (%)	11 (29)	13 (50)	24 (38)
Pancreas histology			
BCA, %	1.14 ± 0.58	0.75 ± 0.34**	0.98 ± 0.53
ACA, %	0.25 ± 0.17	0.22 ± 0.10	0.24 ± 0.15
ACA/BCA	0.24 ± 0.12	0.32 ± 0.14*	0.27 ± 0.14

Data are mean ± SD or *n* (%)

^a^Glycated albumin data were obtained from 18 participants

^b^Timing of blood sampling (i.e. fasting or postprandial) was not determined

^c^Tumour-forming pancreatitis, disseminated sarcoma originating from small intestine, metastatic pancreatic tumour, solid pseudopapillary neoplasm, serous cystic neoplasm, gastrointestinal stromal tumour and gastric cancer; *n* = 1 for each

*p < 0.05, **p < 0.01 vs NDM

IPMN, intraductal papillary mucinous neoplasm

### Measurements and questionnaire

We obtained information about the details of pancreatic diseases, surgical procedures, body height and weight at the time of the operation from medical records. HbA_1c_, glycated albumin and plasma glucose levels were extracted from patients’ blood test data. HbA_1c_ was measured by HPLC (HLC723G11, Tosoh, Tokyo, Japan) and expressed as National Glycohemoglobin Standardization Program value (%) and International Federation of Clinical Chemistry value (mmol/mol). Glycated albumin was measured by an enzymatic method (Lucica GA-L, Sekisui Medical, Tokyo, Japan).

The participants were asked to answer a questionnaire about their weight history and family history of diabetes. The detailed content of the questionnaire was as follows: (1) birthweight; (2) childhood and adolescent (i.e. early childhood, elementary school age, junior high school age and high school age) weight trajectories divided into five categories (i.e. very thin, thin, normal, fat, very fat); (3) body weight at the age of 20 and every decade thereafter; (4) maximum body weight in life; and (5) first- and second-degree family history of diabetes. We defined those who selected ‘fat’ or ‘very fat’ at least once in their childhood and adolescent weight trajectories as having a history of childhood obesity. All participants reached their maximum body weight after 20 years. Adulthood obesity was defined as current BMI of 25 kg/m^2^ or greater [16], and patients whose maximum BMI was 25 kg/m^2^ or greater were defined as having a history of adulthood obesity.

### Pancreatic tissue processing

Pancreatic tissue resected at operation was immediately fixed in formaldehyde and embedded in paraffin for subsequent analysis. Of every four patients who underwent total pancreatectomy, pancreatic head tissues were sampled from two individuals and pancreatic body and tail tissues were sampled from the other two individuals, because of differences in surgical method. Then, 5 μm sections were cut from the tumour-free region and stained for light microscopy as follows: (1) with haematoxylin–eosin; (2) for insulin (peroxidase staining) with haematoxylin; (3) for glucagon with haematoxylin; and (4) for insulin and Ki67 for assessment of beta cell replication, as previously described [8, 9] (ESM Table 1). Antigen retrieval for Ki67 staining was carried out by heat treatment at 120°C in 0.01 mol/l citrate buffer pH 6.0 using an autoclave instrument for 20 min and cooled down to room temperature (RT) and moved into PBS. Quenching of endogenous peroxidase was performed in 0.3% H_2_O_2_ in methanol, for 10 min at RT and rinsed with distilled water and then washed three times with PBS for 5 min. These slides were treated with primary antibodies for 3 h at RT and washed with PBS, then treated with peroxidase-conjugated secondary antibodies for 30 min at RT and rinsed with PBS. Color development was done using treatment with 3,3′-diaminobenzidine (DAB) or SG Peroxidase Substrate Kit SK4700 (Vector, Burlingame, CA, USA). Finally, haematoxylin or nuclear fast red counterstaining was performed.

### Morphometric analysis

To quantify fractional beta cell area (BCA), a single cross-sectional pancreatic section for each participant was used. The entire pancreatic section containing ~300 islets (total pancreas area 126 ± 50 mm^2^) was imaged at the original magnification of ×200 (×20 objective) using a NanoZoomer-XR slide scanner and viewed with NDP.view2 software (Hamamatsu Photonics, Shizuoka, Japan), and the ratio of BCA to total pancreas area was digitally measured using Image Pro Premier software (Media Cybernetics, Silver Spring, MD, USA). Likewise, the ratio of alpha cell area (ACA) to total pancreas area was also digitally measured, and the ratio of ACA to BCA was determined in each case. All measurements were conducted by a single investigator (H. Sasaki); the inter-observer coefficient of variance was assessed between H. Sasaki and other colleagues in the laboratory and was approximately 11%; the intra-observer coefficient of variance was approximately 5%. All measurements were conducted twice, and the mean of the two measurements was used. At the time of the measurement, the investigator was blinded to birthweight, BMI and the glucose metabolism status for each specimen.

To conduct further morphometric analysis, mean islet size and islet density were quantified in randomly selected areas of the pancreas that contained at least 100 islets in each case (105 ± 5 islets, total 6741 islets, ESM Table 2) using NDP.view2 [8, 9]. Furthermore, as surrogate markers of beta cell turnover, scattered beta cells, insulin-positive duct cells and beta cell replication (i.e. by double staining with insulin and Ki67) were quantified. Since the frequency of beta cell apoptosis was very low, as observed in our prior reports [3, 8, 9], it was not evaluated in this study. Scattered beta cells were defined as a cluster of three or fewer beta cells in acinar tissue, and the density of scattered beta cells was determined as the number of scattered beta cells/pancreas area (/mm^2^). Insulin-positive duct cells were also counted and expressed as the number of insulin-positive duct cells/pancreas area (/mm^2^). Frequency of beta cell replication was expressed as the percentage of islets. Mean beta cell diameter was measured as a surrogate of beta cell size, as previously described [8].

### Statistical analysis

Data are presented as mean ± SD in the text and tables unless otherwise indicated. Mann–Whitney *U* test was used to analyse the difference between two groups, and Spearman correlation coefficient was used to assess the correlation between two variables. All analyses were performed using SPSS (version 25; SPSS, IBM, Chicago, IL, USA). A value of *p* < 0.05 was taken as statistically significant.

## Results

### Participant characteristics

Characteristics of participants with (DM group, *n* = 26) and without diabetes (NDM group, *n* = 38) are shown in Table 1 and ESM Table 2. There was no significant difference in age between the two groups. In the DM group, HbA_1c_, glycated albumin and plasma glucose levels, and current and maximum BMI, were significantly higher than in the NDM group. In both groups, pancreatic cancer was the most common pancreatic disease and pancreatoduodenectomy was the most frequent operative procedure. Mean birthweight was 3026 ± 466 g, and there was no significant difference between the groups. In the NDM and DM groups, 47% and 92% of patients, respectively, had a history of obesity during their life, and 16% and 35%, respectively, had a history of childhood obesity.

### Effects of diabetes on islet morphology

BCA was significantly smaller in the DM group compared with the NDM group (0.75 ± 0.34% vs 1.14 ± 0.58%, *p* < 0.01, Table 1, ESM Fig. 1). There was no difference in ACA between the two groups (0.22 ± 0.10% vs 0.25 ± 0.17%, *p* = 0.98). Thus, the ACA to BCA ratio in the DM group was higher than that in the NDM group (0.32 ± 0.14 vs 0.24 ± 0.12, *p* = 0.01).

Islet density was significantly reduced in the DM group compared with the NDM group (2.69 ± 1.07 vs 3.90 ± 1.96 /mm^2^, *p* < 0.01, ESM Fig. 1). Likewise, the DM group showed significantly smaller mean islet size than the NDM group (4505 ± 1459 vs 7052 ± 2663 μm^2^, *p* < 0.01).

### Birthweight and beta and alpha cell mass

**Fig. 1:**
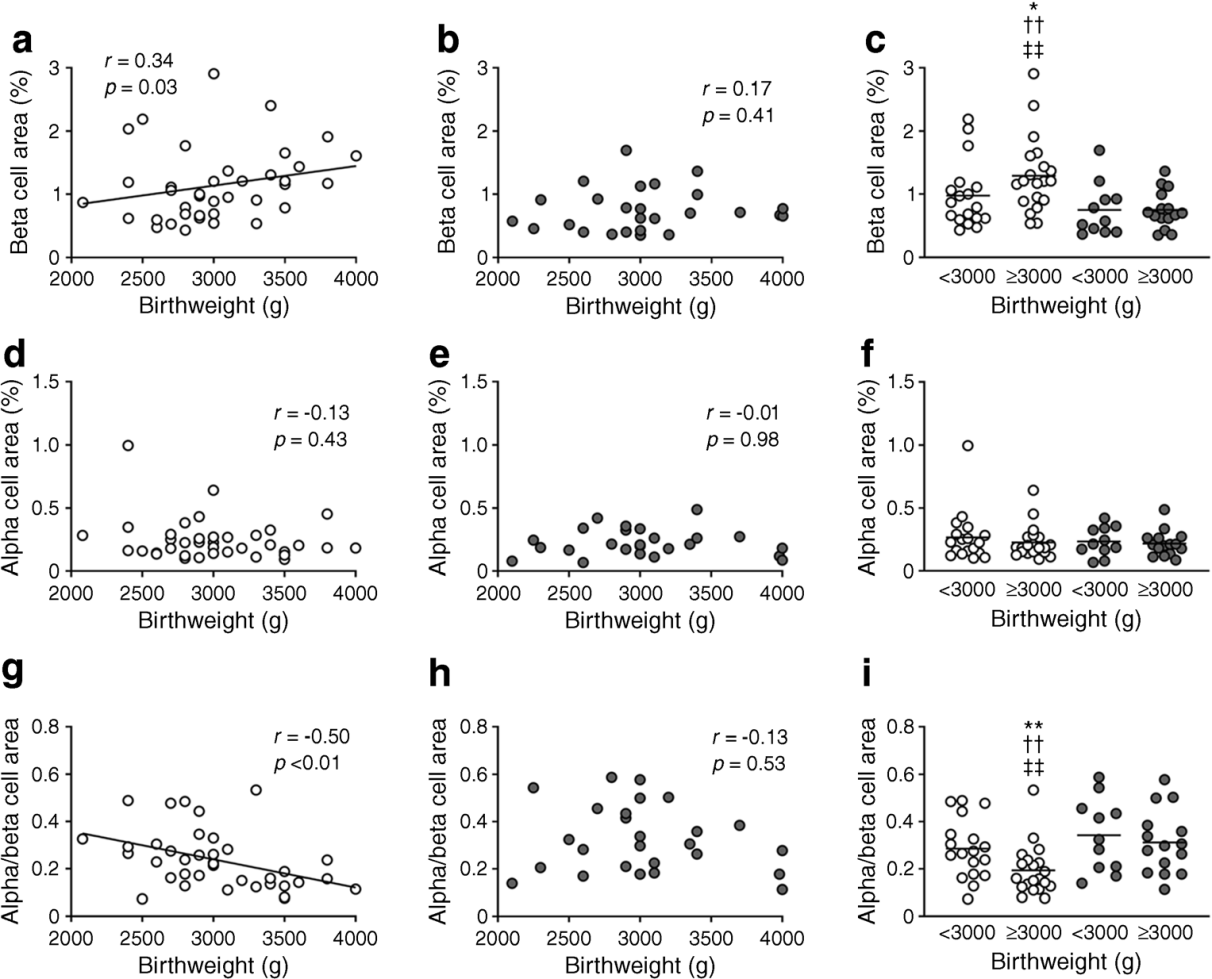
Correlation between birthweight and BCA (**a**–**c**), ACA (**d**–**f**), or ACA to BCA ratio (**g**–**i**) in participants with (DM group) and without (NDM group) diabetes. Grey and white circles show DM and NDM participants, respectively. Bars indicate mean. *p < 0.05, **p < 0.01 vs NDM participants with birthweight <3000 g; ^††^*p* < 0.01 vs DM participants with birthweight <3000 g; ^‡‡^*p* < 0.01 vs DM participants with birthweight ≥3000 g

In the NDM group, there was a weak positive correlation between birthweight and BCA (*R* = 0.34, *p* = 0.03, Fig. 1), but no correlation between birthweight and ACA (*R* = −0.13, *p* = 0.43). Thus, a negative correlation was observed between birthweight and ACA to BCA ratio in the NDM group (*R* = −0.50, *p* < 0.01). However, in the DM group, there was no correlation between birthweight and BCA, ACA, or ACA to BCA ratio (Fig. 1).

The NDM and DM groups were further classified according to the mean birthweight (Fig. 1, ESM Table 3). In the NDM group, those with birthweight ≥3000 g showed greater BCA than those with birthweight <3000 g (1.29 ± 0.60% vs 0.98 ± 0.53%, *p* = 0.04), with no significant difference in individual beta cell size (ESM Fig. 2).

### Birthweight and other islet morphology

**Fig. 2:**
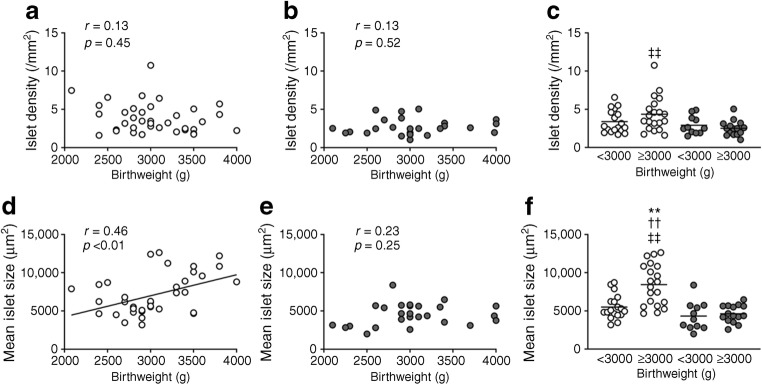
Correlation between birthweight and islet density (**a**–**c**) or mean islet size (**d**–**f**) in participants with (DM group) and without (NDM group) diabetes. Grey and white circles show DM and NDM participants, respectively. Bars indicate mean. **p < 0.01 vs NDM participants with birthweight <3000 g; ^††^*p* < 0.01 vs DM participants with birthweight <3000 g; ^‡‡^*p* < 0.01 vs DM participants with birthweight ≥3000 g

In the NDM group, birthweight and islet density were not correlated (*R* = 0.13, *p* = 0.45, Fig. 2), while there was a positive correlation between birthweight and mean islet size (*R* = 0.46, *p* < 0.01). Neither islet density nor mean islet size and birthweight were correlated in the DM group (*R* = 0.13, *p* = 0.52 and *R* = 0.23, *p* = 0.25, respectively). The NDM group with birthweight ≥3000 g showed greater mean islet size (8441 ± 2692 vs 5510 ± 1595 μm^2^, *p* < 0.01), but not islet density, than the NDM group with birthweight <3000 g.

**Fig. 3:**
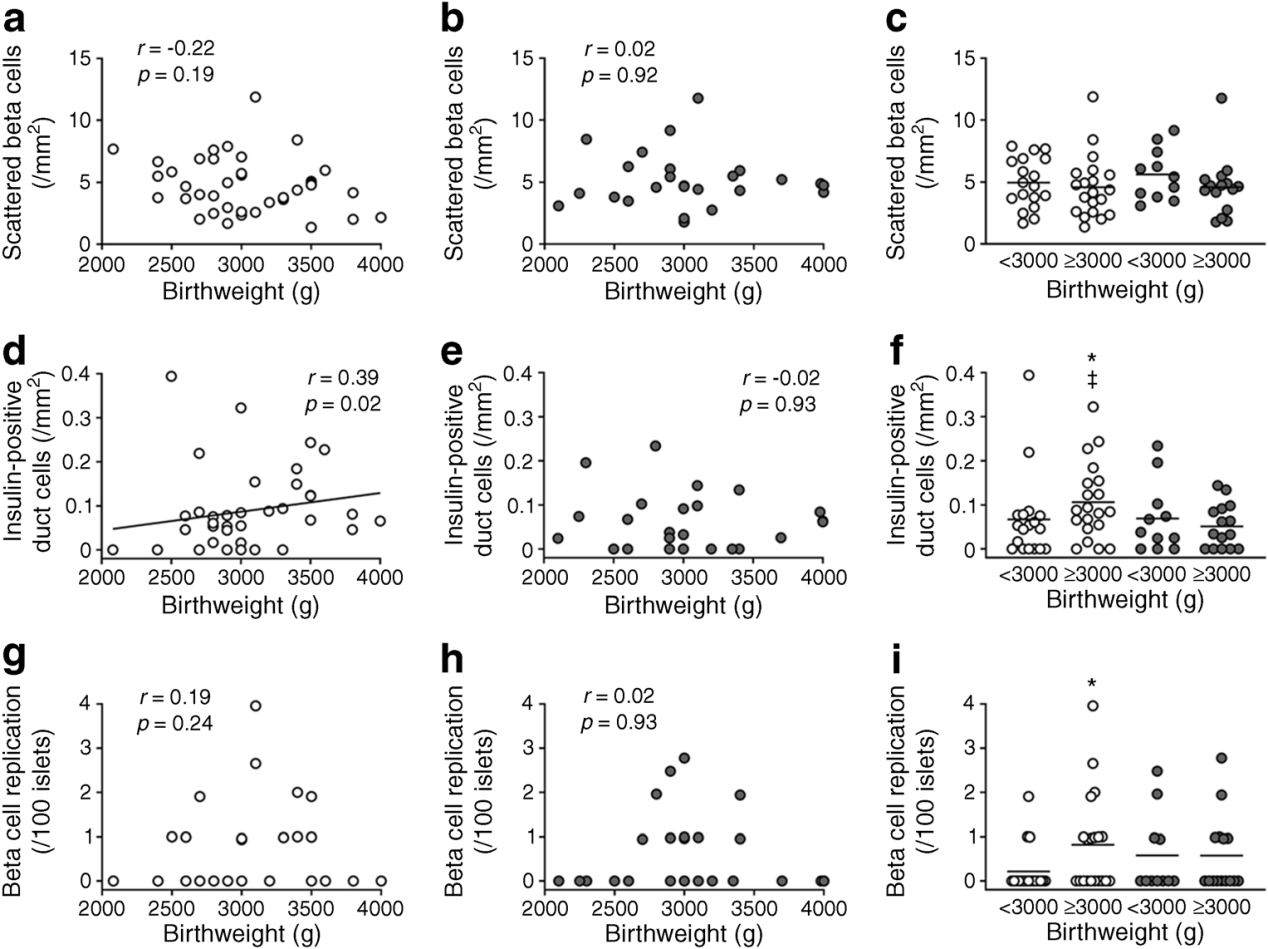
Correlation between birthweight and number of scattered beta cells (**a**–**c**), number of insulin-positive duct cells (**d**–**f**) and beta cell replication (**g**–**i**) in participants with (DM group) and without (NDM group) diabetes. Grey and white circles show DM and NDM participants, respectively. Bars indicate mean. *p < 0.05 vs NDM participants with birthweight <3000 g; ^‡^*p* < 0.05 vs DM participants with birthweight ≥3000 g

Regarding surrogate markers of beta cell turnover (ESM Fig. 2), a positive correlation was found between birthweight and density of insulin-positive duct cells in the NDM group (*R* = 0.39, *p* = 0.02, Fig. 3). The NDM group with birthweight ≥3000 g showed an increase in density of insulin-positive duct cells (0.11 ± 0.09 vs 0.07 ± 0.10 /mm^2^, *p* = 0.04) and frequency of beta cell replication (0.82 ± 1.09 vs 0.22 ± 0.53 /100 islets, *p* = 0.04) compared with the NDM group with birthweight <3000 g.

### Effects of current and childhood obesity on islet morphology

There was no correlation between current BMI and islet morphology including BCA, ACA, ACA to BCA ratio, islet density and mean islet size in either the NDM or DM group (ESM Fig. 3). Similarly, maximum BMI and islet morphology were not correlated in either group (ESM Fig. 4).

**Fig. 4:**
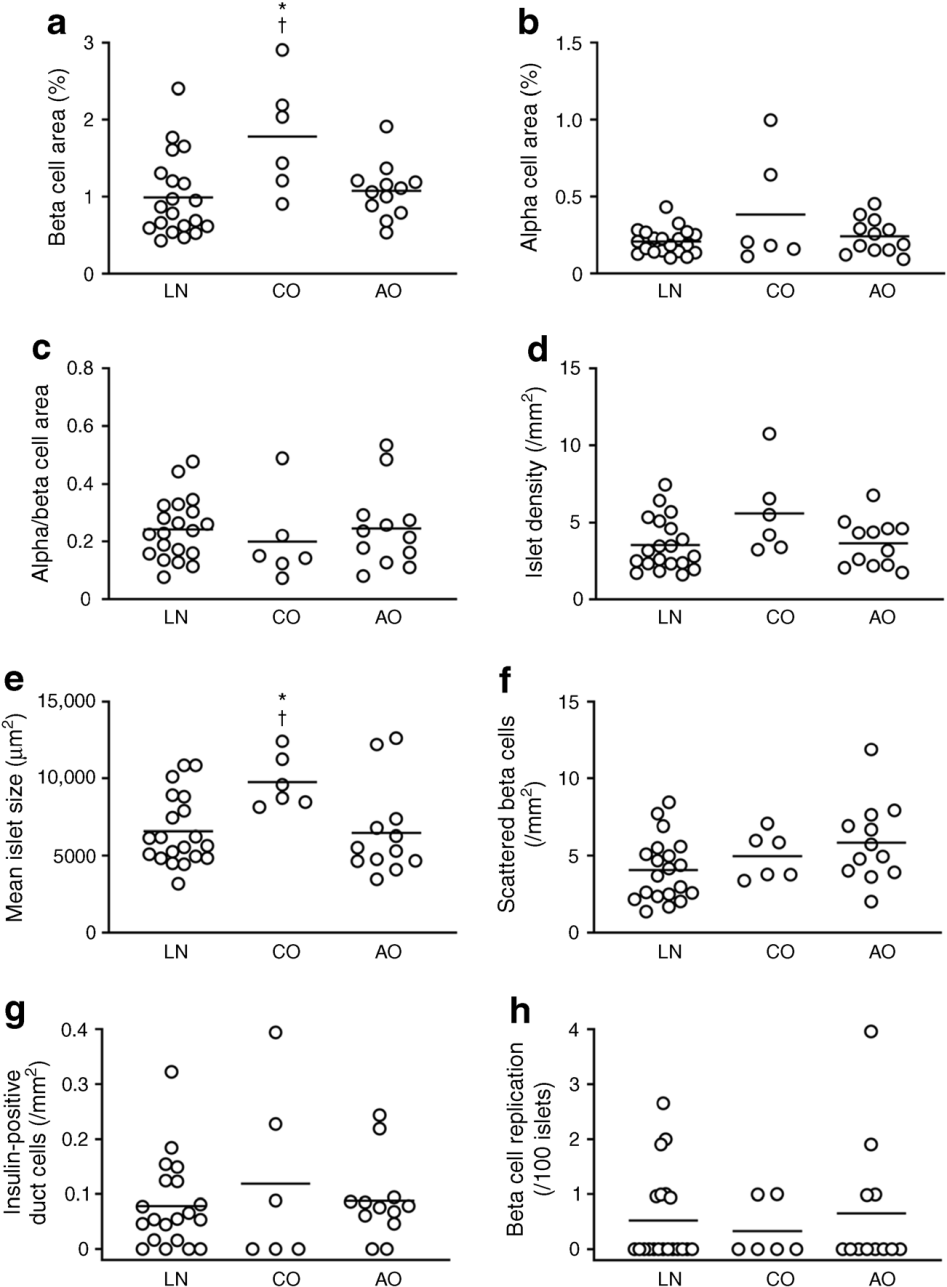
Effects of history of childhood obesity and adulthood obesity on BCA (**a**), ACA (**b**), ACA to BCA ratio (**c**), islet density (**d**), mean islet size (**e**) and beta cell turnover (**f**–**h**) in participants without diabetes (NDM group). LN, NDM-LN group; CO, NDM-CO group; AO, NDM-AO group. Bars indicate mean. *p < 0.05 vs NDM-LN; ^†^*p* < 0.05 vs NDM-AO

To explore the effects of childhood obesity, the NDM group was divided into three categories, i.e. participants with a history of childhood obesity (NDM-CO group, *n* = 6), those with a history of obesity only in adulthood (NDM-AO group, *n* = 12) and those with no history of obesity (lean; NDM-LN group, *n* = 20) (Fig. 4, ESM Table 4). The NDM-CO group, but not the NDM-AO group, showed greater BCA than the NDM-LN group (1.78 ± 0.74% vs 0.99 ± 0.53%, *p* = 0.01). The NDM-CO, but not NDM-AO, group also showed greater mean islet size compared with the NDM-LN group (9759 ± 1716 vs 6586 ± 2259 μm^2^, *p* = 0.01). In the NDM-CO group, no correlation was observed between birthweight and BCA (*R* = −0.60, *p* = 0.21, ESM Fig. 5), and there was no difference in BCA (1.89 ± 0.40% vs 1.67 ± 1.08%, *p* = 0.51) or mean islet size (8919 ± 585 vs 10,599 ± 2215 μm^2^, *p* = 0.51) between participants with and without adulthood obesity (*n* = 3, respectively). Of note, there was no difference in birthweight among the groups (ESM Table 4).

By contrast, in the DM group, when similarly classified by history of obesity, no difference in islet morphology was observed among the three groups (ESM Fig. 6, ESM Table 5), although there were only two participants in the DM-LN group.

### Correlation coefficient between birthweight and BCA was increased in participants without a history of childhood obesity

**Fig. 5:**
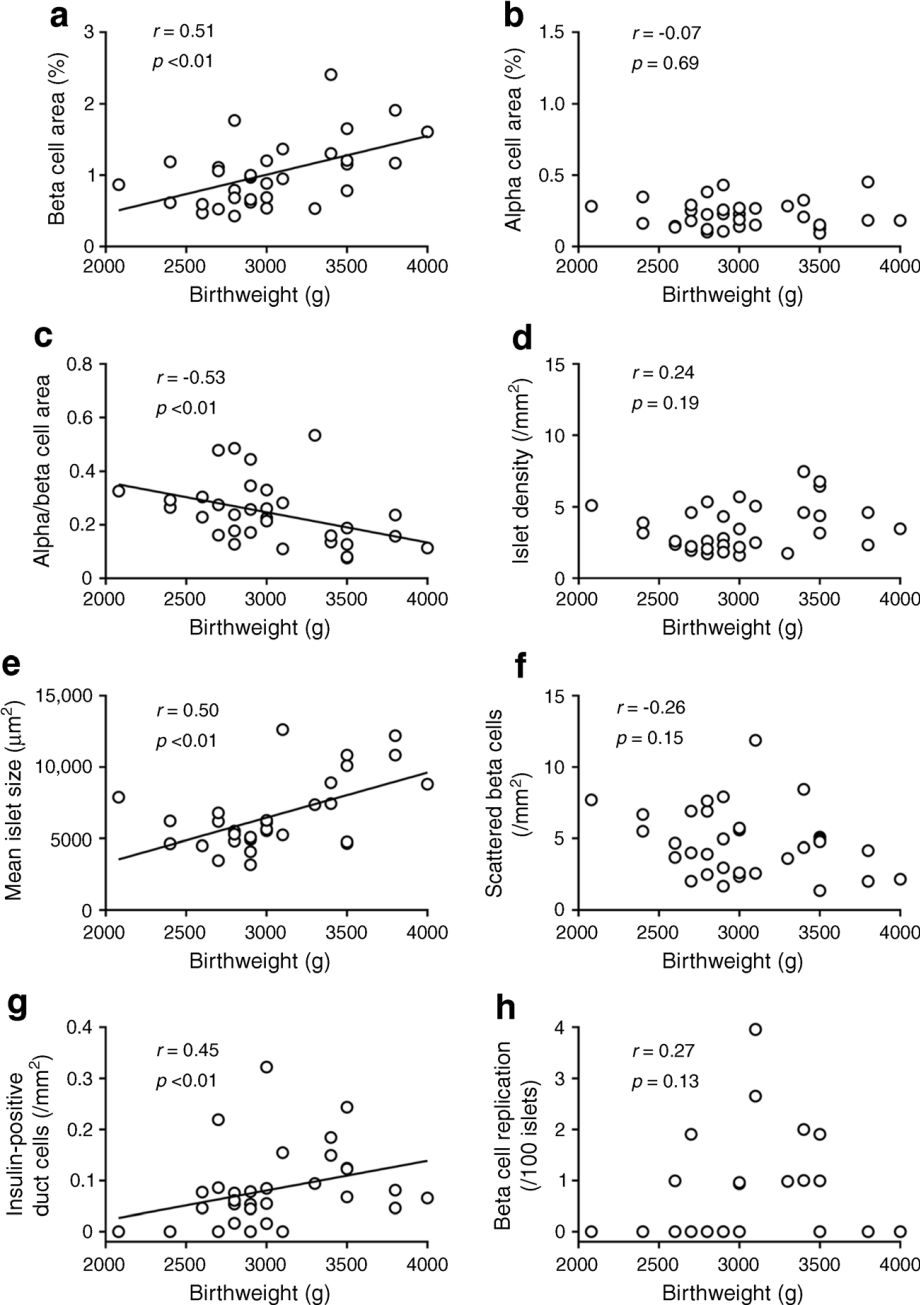
Correlation between birthweight and BCA (**a**), ACA (**b**), ACA to BCA ratio (**c**), islet density (**d**), mean islet size (**e**) and beta cell turnover (**f**–**h**) in NDM participants without a history of childhood obesity (NDM-LN and NDM-AO groups; *n* = 32)

Finally, considering the possibility that a history of childhood obesity may affect BCA in the NDM group, we further analysed the association between birthweight and BCA after excluding the NDM-CO group (*n* = 32, Fig. 5). As a result, the positive correlation coefficient between birthweight and BCA was increased (*R* = 0.51, *p* < 0.01). The positive correlation coefficients between birthweight and mean islet size and the density of insulin-positive duct cells, and the negative correlation coefficient between birthweight and ACA to BCA ratio were also increased (*R* = 0.50, 0.45 and −0.53, respectively, all *p* < 0.01). However, there remained no correlation between birthweight and ACA, islet density, density of scattered beta cells or frequency of beta cell replication. The positive correlation between birthweight and BCA was observed in both sexes (ESM Fig. 7). These results were consistent when only the NDM-LN group was analysed (*n* = 20, ESM Fig. 8).

## Discussion

In this study, we report that: (1) birthweight and BCA, but not ACA, were positively correlated in Japanese non-diabetic adults; (2) in these individuals, birthweight and mean islet size, but not islet density, were positively correlated; (3) these correlations were not observed in participants with type 2 diabetes; and (4) a history of childhood obesity, rather than adulthood obesity, may affect BCA and mean islet size.

Asians are thought to have a lower insulin secretion capacity compared with people of European or African descent [17]. In previous studies, including ours, neither current BMI nor maximum BMI in life were correlated with BCA in Japanese adults [3, 8, 18, 19]. The same results were also observed in this study, suggesting that Asians lack the capacity for beta cell proliferation to compensate for insulin resistance, compared with other ethnicities [1]. However, these previous studies only took into account obesity in adulthood.

Although there are conflicting results regarding the association between high birthweight and incidence of type 2 diabetes [20–22], infants with low birthweight have been consistently reported to have increased incidence of type 2 diabetes [12, 23–31], which has been recently proposed as part of the DOHaD hypothesis [13, 14]. Although there is complex gene–environment interaction and the mechanisms of the association between low birthweight and future development of type 2 diabetes remain uncertain, an involvement of epigenetic modification has been recently proposed [32, 33]. Recent evidence has suggested that fetal undernutrition causes epigenetic modifications including DNA methylation, histone modifications and microRNA interaction, inducing beta cell dysfunction and insulin resistance in the offspring [32, 33]. It has been shown that mice with low birthweight are susceptible to impaired glucose tolerance due to epigenetic control, which suppresses beta cell proliferation [34]. In this study, we found that birthweight and BCA were positively correlated in Japanese non-diabetic adults, indicating that birthweight is a major determinant of BCM not only in rodents but also in humans.

Obesity in adulthood is a well-known risk for the development of type 2 diabetes [35]. Although individuals with low birthweight are at risk of developing type 2 diabetes, it has been reported that those born with low birthweight and with obesity in adulthood showed the highest risk of type 2 diabetes compared with those with normal birthweight and without adulthood obesity [36, 37], suggesting a significant interaction between birthweight and adulthood obesity in the incidence of type 2 diabetes. Taken together, our findings suggest that smaller BCM in individuals born with low birthweight explains at least in part the relationship between low birthweight and high risk of future development of type 2 diabetes.

On the other hand, there was no correlation between birthweight and ACA in both non-diabetic and diabetic participants, suggesting that, unlike beta cells, alpha cells may be less susceptible to epigenetic effects of low birthweight. Nonetheless, a negative correlation was observed between birthweight and ACA to BCA ratio in non-diabetic participants, indicating that people born with low birthweight show a relative increase in ACM. Future studies will be needed to explore whether the likelihood of developing type 2 diabetes in infants with low birthweight is more attributable to a decrease in BCM or a relative increase in ACM.

The second finding of this study was clarification of the associations of birthweight with islet density and mean islet size. Analysis of fetal pancreatic tissues has shown that lobular organisation of the pancreas as well as increase in islet size is observed from early prenatal stages [38]. A previous report has shown that BCM and islet size increase from birth to 20 years of age, but density of islets decreases with age [10], suggesting that new beta cell formation mainly occurs with increasing islet size. Here we found a significant correlation between birthweight and mean islet size, but not islet density, in non-diabetic individuals without a history of childhood obesity, suggesting that insufficient development of each islet rather than reduced number of islets is the mechanism of reduced BCM in people born with low birthweight.

Islet development involves beta cell replication, which is highest in neonates up to infancy and decreases with age [10, 11]. In this study, although beta cell replication was very rarely observed in adult humans, the frequency of beta cell replication was significantly lower in those with birthweight <3000 g than in those with birthweight ≥3000 g, suggesting the involvement of beta cell replication in greater islet size. Another source of beta cells may be insulin-positive duct cells, whose number was positively correlated with birthweight in this study. Although beta cell neogenesis and proliferation are active in the neonate, duct cells are assumed to have the capacity to convert to beta cells even in adulthood [39, 40]. Taken together, our findings suggest the possibility that the ability of beta cell production declines during the life course in people born with low birthweight, also highlighting the role of epigenetic modification, which is relatively stably transmitted until adulthood.

The third finding was that no correlation between birthweight and BCA or mean islet size was observed in participants with type 2 diabetes. Previous reports have shown that BCM declines in type 2 diabetes [1–4]. However, it is not yet known whether the decline in BCM in individuals with type 2 diabetes is a cause or consequence of the disease in humans. Our findings indicate that the presence of type 2 diabetes modifies the relationship between birthweight and BCM, and therefore birthweight is no longer a major determinant of BCM in individuals with type 2 diabetes.

Finally, we confirmed the importance of childhood obesity as a factor affecting islet morphology. Intriguingly, compared with non-diabetic participants who did not have a history of childhood obesity, regardless of the presence or absence of adulthood obesity, those with a history of childhood obesity showed significantly larger BCA and mean islet size despite there being no significant difference in birthweight. Furthermore, the correlation coefficients between birthweight and BCA, mean islet size, and density of insulin-positive duct cells in non-diabetic participants were further increased by excluding those with childhood obesity. Although the number of individuals with childhood obesity was small in this study, these results suggest that a history of childhood obesity is a modifier of BCM and islet size. It has been shown that elevated BMI only in adulthood rather than in adolescence was associated with development of diabetes [41]. Another study of Japanese female nurses has also reported an inverse relationship between BMI at age 18 and the incidence of diabetes [42]. Furthermore, adolescent low BMI is associated with an increase in the incidence of gestational diabetes [43, 44]. The inverse relationship between childhood obesity and diabetes may be in part explained by the capacity of beta cell expansion during this age, although it should be noted that an increase in body weight during childhood remains correlated with an increased risk of type 2 diabetes, especially in those with low birthweight [45]. In addition, whether the poor response of BCM to obesity in Japanese is attributable to the lower incidence of childhood obesity in this population [46, 47] is an intriguing question that needs further clarification.

A limitation of this study is that birthweight and history of obesity were based on a questionnaire. The average age of participants in this study was over 60 years, and we were not able to obtain documented records in childhood. However, previous studies with respect to birthweight also used questionnaires to collect information [25–27, 36, 37, 42, 43]. Moreover, we sought to maintain the reliability of the data by including only those who were able to recall their birthweight on a scale of at least every 100 g. Second, different portions of the pancreas were sampled according to the operative procedure. However, the proportion of endocrine cells has been shown to be relatively consistent regardless of the pancreatic site, except for the ventral portion of the pancreatic head [4]. Furthermore, the results were not changed when we analysed the cases of pancreatic head and body/tail separately (ESM Fig. 9). Third, pancreatic diseases might affect islet morphology; however, the results did not change depending on whether or not the primary disease was pancreatic cancer in this study (ESM Fig. 10). In addition, in this study, because of the presence of pancreatic diseases we were not able to measure pancreas weight or volume, and therefore actual BCM, the product of BCA and pancreas weight, was undetermined, although BCA is widely used as a surrogate for BCM. Thus, difference in pancreas weight might affect our findings, as reduced pancreas weight has been reported in animal models of intrauterine growth retardation [48]. Nonetheless, these limitations and biases described above should make the correlations or differences between the groups tend towards zero. Fourth, since all participants of this study were Japanese, our findings may not be applicable to other ethnicities. Finally, in our participants, there were only seven participants with birthweight <2500 g, which is generally considered as low birthweight [21], furthermore, owing to the nature of the cross-sectional design of the study, it is unclear whether those with low BCM would develop type 2 diabetes in the future. Our findings should be confirmed by future studies including a larger sample size and multiple ethnicities.

In conclusion, there was a positive correlation between birthweight and BCM, and birthweight and mean islet size in Japanese non-diabetic adults. ACM and islet density were not related to birthweight. A history of childhood obesity and the presence of type 2 diabetes may be factors affecting BCM after birth. These findings will be important bases for explaining the DOHaD hypothesis.

## Electronic supplementary material

ESM (PDF 1.64 mb)

## Data Availability

The datasets generated during and/or analysed during the current study are available from the corresponding author on reasonable request.

## Abbreviations

ACA: Alpha cell area
ACM: Alpha cell mass
BCA: Beta cell area
BCM: Beta cell mass
DM (group): Participant group with diabetes
DOHaD: Developmental Origins of Health and Disease
NDM (group): Participant group without diabetes
NDM-AO (group): NDM group with history of obesity only in adulthood
NDM-CO (group): NDM group with history of childhood obesity
NDM-LN (group): NDM group with no history of obesity (lean)
