# Supplementary material for: Associations of birthweight and history of childhood obesity with beta cell mass in Japanese adults
Source: Diabetologia. 2020 Apr 1;63(6):1199–210. doi: 10.1007/s00125-020-05127-2 (PMC7228916; doi:10.1007/s00125-020-05127-2)
Supplement: Supplementary file 1 — (PDF 1.64 mb) [file 125_2020_5127_MOESM1_ESM.pdf]

ESM Table 1. Antibodies used in this study.

Primary antibody					Secondary antibody	
Antigen	Code	Company	Dilution	Species	Code	Company
Insulin	A0564	Dako	1:500	Guinea pig, polyclonal	P0141	Dako
Glucagon	A0565	Dako	1:1000	Rabbit, polyclonal	MP7401	Vector
Ki67	M7240	Dako	1:200	Mouse, monoclonal	MP7402	Vector

ESM Table 2. Characteristics of each participant.

No	Age, years	Sex (M: Male/ F: Female)	Current BMI, kg/m^2	Maximum BMI, kg/m^2	Group	Period of childhood obesity	Diabetes treatment before operation	Total pancreas area, mm^2	Number of islets counted for islet density and size
1	72	F	22.6	23.6	NDM-LN	-	-	119.1	107
2	44	F	21.3	22.1	NDM-LN	-	-	78.7	105
3	78	F	17.7	21.9	NDM-LN	-	-	90.9	104
4	81	F	15.7	21.6	NDM-LN	-	-	94.8	105
5	51	F	19.1	20.5	NDM-LN	-	-	95.4	113
6	71	F	19.0	23.5	NDM-LN	-	-	140.0	102
7	47	F	20.4	21.9	NDM-LN	-	-	277.0	103
8	63	F	17.8	23.5	NDM-LN	-	-	184.3	104

9	22	F	23.0	23.1	NDM-LN	-	-	113.6	102
10	62	M	22.9	23.3	NDM-LN	-	-	130.9	101
11	62	M	20.9	24.9	NDM-LN	-	-	97.1	113
12	64	F	18.0	20.6	NDM-LN	-	-	72.3	103
13	68	M	19.8	21.5	NDM-LN	-	-	44.4	100
14	53	M	22.7	23.2	NDM-LN	-	-	152.0	101
15	70	F	18.1	18.8	NDM-LN	-	-	31.4	100
16	62	F	18.8	23.2	NDM-LN	-	-	129.4	108
17	68	F	17.9	18.2	NDM-LN	-	-	133.0	100
18	75	F	16.5	22.4	NDM-LN	-	-	128.7	105
19	56	M	21.8	23.5	NDM-LN	-	-	117.5	102
20	32	M	22.9	23.2	NDM-LN	-	-	219.7	106
21	56	F	22.7	23.5	NDM-CO	Junior high school	-	116.2	102
22	55	M	27.1	37.4	NDM-CO	Elementary, junior high and	-	70.8	110

						high school			
23	35	M	22.0	22.2	NDM-CO	Early childhood and elementary school	-	90.0	110
24	57	M	32.0	33.6	NDM-CO	Junior high and high school	-	145.2	104
25	80	F	25.7	32.7	NDM-CO	Early childhood, elementary, junior high and high school	-	107.1	100
26	69	M	24.9	24.9	NDM-CO	Elementary school	-	130.2	101
27	38	M	24.1	25.7	NDM-AO	-	-	129.0	100
28	66	M	25.5	27.7	NDM-AO	-	-	84.1	100
29	77	F	26.2	26.2	NDM-AO	-	-	66.2	103
30	67	M	24.5	26.6	NDM-AO	-	-	149.1	104

31	78	F	24.4	26.1	NDM-AO	-	-	141.5	105
32	78	M	26.4	30.4	NDM-AO	-	-	211.7	108
33	58	M	25.7	26.3	NDM-AO	-	-	184.1	104
34	55	M	28.3	29.4	NDM-AO	-	-	175.3	111
35	53	M	22.2	25.1	NDM-AO	-	-	57.1	100
36	79	M	24.4	28.3	NDM-AO	-	-	150.0	100
37	76	M	25.8	28.5	NDM-AO	-	-	146.4	102
38	67	M	19.9	26.4	NDM-AO	-	-	89.8	111
39	70	M	20.2	23.2	DM-LN	-	Diet therapy	73.5	104
40	71	M	19.9	19.9	DM-LN	-	DPP-4i [a], SU [b]	142.5	102
41	62	M	26.0	33.5	DM-CO	Early childhood, elementary, junior high and high school	α-GI [c], SU	108.6	117

42	72	M	19.2	27.4	DM-CO	High school	Diet therapy	92.6	102
43	47	M	20.7	29.4	DM-CO	Junior high and high school	Insulin	265.5	102
44	70	M	29.0	32.3	DM-CO	Early childhood, elementary, junior high and high school	α-GI, DPP-4i, TZD [d]	141.2	116
45	39	M	28.1	37.1	DM-CO	Early childhood, elementary, junior high and high school	α-GI, DPP-4i, SU	127.2	103
46	85	F	22.9	27.6	DM-CO	Early childhood and elementary school	DPP-4i	116.1	111
47	63	M	22.5	25.5	DM-CO	High school	DPP-4i,	94.4	105

							Met [e], SU		
48	61	M	28.5	32.3	DM-CO	High school	Insulin	73.7	105
49	71	M	24.6	27.1	DM-CO	High school	Diet therapy	59.3	103
50	43	M	23.1	28.2	DM-AO	-	Insulin	160.1	107
51	65	M	27.5	28.6	DM-AO	-	Met	160.5	104
52	77	M	21.5	26.0	DM-AO	-	DPP-4i, Insulin	39.5	105
53	65	M	25.0	25.9	DM-AO	-	Diet therapy	196.8	116
54	75	M	27.9	28.3	DM-AO	-	α-GI, DPP-4i, Glinide	109.4	121
55	73	F	25.2	26.0	DM-AO	-	Glinide	197.6	106
56	69	F	32.8	32.8	DM-AO	-	Diet therapy	137.5	110
57	81	M	29.9	36.2	DM-AO	-	α-GI, DPP-4i, SU	108.5	105

58	78	M	26.4	30.9	DM-AO	-	DPP-4i, Met, TZD	92.0	101
59	70	M	26.8	26.8	DM-AO	-	Insulin, Met, SGLT2i [f]	142.2	104
60	74	M	26.4	28.7	DM-AO	-	Diet therapy	113.4	103
61	72	M	27.5	27.7	DM-AO	-	α-GI, Met	92.8	101
62	72	M	21.4	26.0	DM-AO	-	DPP-4i	168.7	111
63	71	M	21.7	26.0	DM-AO	-	Insulin	158.1	108
64	51	M	27.1	27.1	DM-AO	-	Diet therapy	193.6	110

a DPP-4i: Dipeptidyl peptidase-4 inhibitor

b SU: Sulfonylurea

c α-GI: Alpha-glucosidase inhibitor

d TZD: Thiazolidine

e Met: Metformin

f SGLT2i: SGLT2 inhibitor

ESM Table 3. Characteristics of participants according to birth weight of under 3,000 g and over 3,000 g.

	Patients without diabetes (NDM)		Patients with diabetes (DM)	
Categories of birth weight	<3,000 g	≥3,000 g	<3,000 g	≥3,000 g
N (male/female)	18 (8/10)	20 (12/8)	11 (8/3)	15 (15/0)
Age, years	64.8 ± 10.5	58.9 ± 16.9	71.4 ± 9.8 [b]	64.1 ± 11.5
Current BMI, kg/m^2	21.8 ± 3.5	22.8 ± 3.7	25.4 ± 4.1 [a]	24.8 ± 3.1 [a]
Maximum BMI, kg/m^2	25.2 ± 4.5	24.6 ± 3.6	28.8 ± 3.1 [c, d]	28.3 ± 4.4 [d]
HbA1c, mmol/mol	39 ± 4	37 ± 5	52 ± 7 [c, d]	51 ± 12 [c, d]
HbA1c, %	5.8 ± 0.4	5.6 ± 0.5	6.9 ± 0.7 [c, d]	6.8 ± 1.2 [c, d]
Birth weight, g	2,660 ± 228	3,350 ± 303 [c, e]	2,595 ± 281	3,349 ± 390 [c, e]

Data are mean ± SD or n.

a *P*<0.05 vs. NDM group with birth weight under 3,000 g

b *P*<0.05 vs. NDM group with birth weight over 3,000 g

c $P<0.01$ vs. NDM group with birth weight under 3,000 g

d $P<0.01$ vs. NDM group with birth weight over 3,000 g

e $P<0.01$ vs. DM group with birth weight under 3,000 g

ESM Table 4. Characteristics of participants according to obesity history in NDM group.

	NDM-LN	NDM-CO	NDM-AO
N (male/female)	20 (6/14)	6 (4/2)	12 (10/2)
Age, years	60.1 ± 15.1	58.7 ± 15.1	66.0 ± 12.9
Current BMI, kg/m^2	19.8 ± 2.3	25.8 ± 3.6 [a]	24.8 ± 2.2 [a]
Maximum BMI, kg/m^2	22.2 ± 1.7	29.1 ± 6.3 [a]	27.2 ± 1.6 [a]
HbA1c, mmol/mol	39 ± 4	38 ± 3	37 ± 6
HbA1c, %	5.7 ± 0.4	5.7 ± 0.3	5.6 ± 0.6
Birth weight, g	3,019 ± 470	3,000 ± 469	3,042 ± 410

Data are mean ± SD or n.

a $P<0.01$ vs. NDM-LN

NDM-LN: No history of obesity in childhood or adulthood

NDM-CO: History of childhood obesity regardless of presence or absence of adulthood obesity

NDM-AO: No history of childhood obesity but history of adulthood obesity

ESM Table 5. Characteristics of participants according to obesity history in DM group.

	DM-LN	DM-CO	DM-AO
N (male/female)	2 (2/0)	9 (8/1)	15 (13/2)
Age, years	70.5 ± 0.7	63.3 ± 13.8	69.1 ± 10.1
Current BMI, kg/m^2	20.0 ± 0.2	24.6 ± 3.6	26.0 ± 3.2 [a]
Maximum BMI, kg/m^2	21.5 ± 2.3	30.2 ± 3.8 [a]	28.3 ± 2.9 [a]
HbA1c, mmol/mol	48 ± 23	56 ± 13	49 ± 6
HbA1c, %	6.6 ± 2.2	7.3 ± 1.2	6.6 ± 0.6
Birth weight, g	3,050 ± 71	3,198 ± 516	2,927 ± 533

Data are mean ± SD or n.

a P<0.05 vs. DM-LN

DM-LN: No history of obesity in childhood or adulthood

DM-CO: History of childhood obesity regardless of presence or absence of adulthood obesity

DM-AO: No history of childhood obesity but history of adulthood obesity

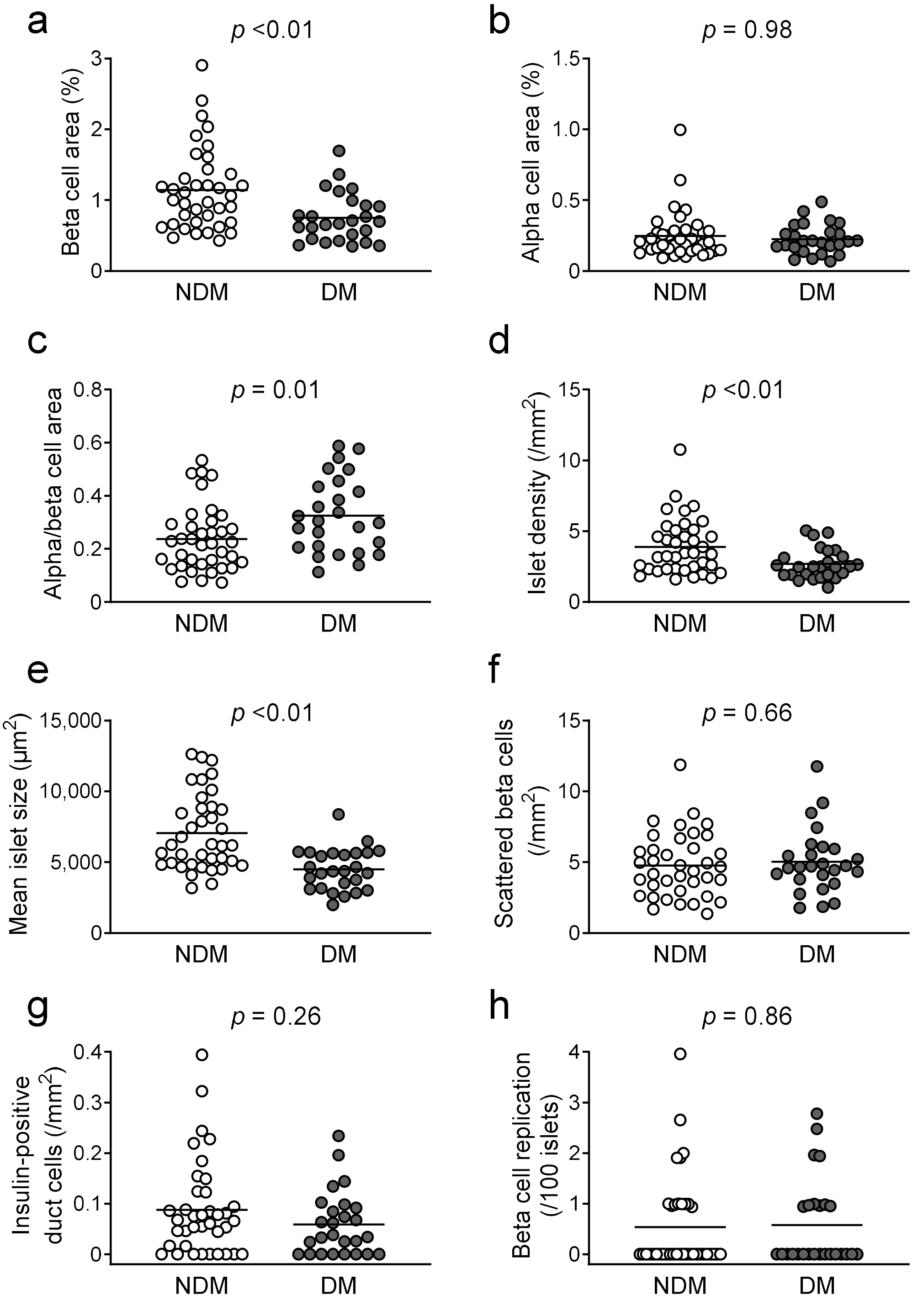

ESM Figure 1.

Effects of diabetes on beta cell area (BCA, a), alpha cell area (ACA, b), ACA to BCA ratio (c), islet density (d), mean islet size (e) and beta cell turnover (f-h). Grey and white circles show participants with (DM group) and without (NDM group) diabetes, respectively. Bars indicate mean.

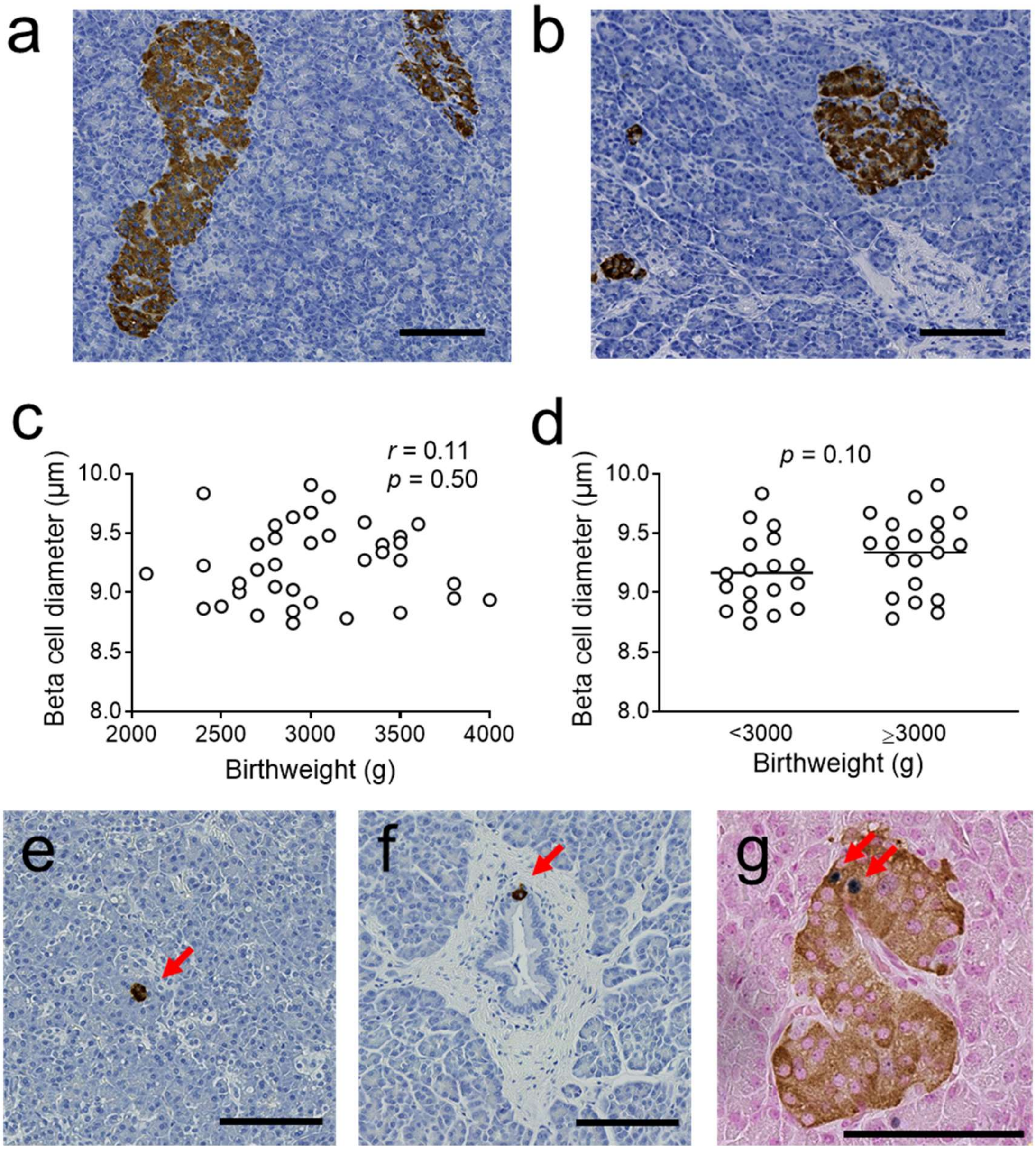

ESM Figure 2.

Representative photographs of pancreas immunostained for insulin (brown). Examples of non-diabetic cases born with normal birth weight (3,200 g, a) and low birth weight (2,400 g, b). Correlation between birth weight and individual beta cell size (mean beta cell diameter) in non-diabetic participants (c and d). Representative photographs of beta cell turnover. Scattered beta cells (e), insulin-positive duct cells (f) and beta cell replication i.e., double staining of insulin and Ki67 (g). Scale bar, 100 μm.

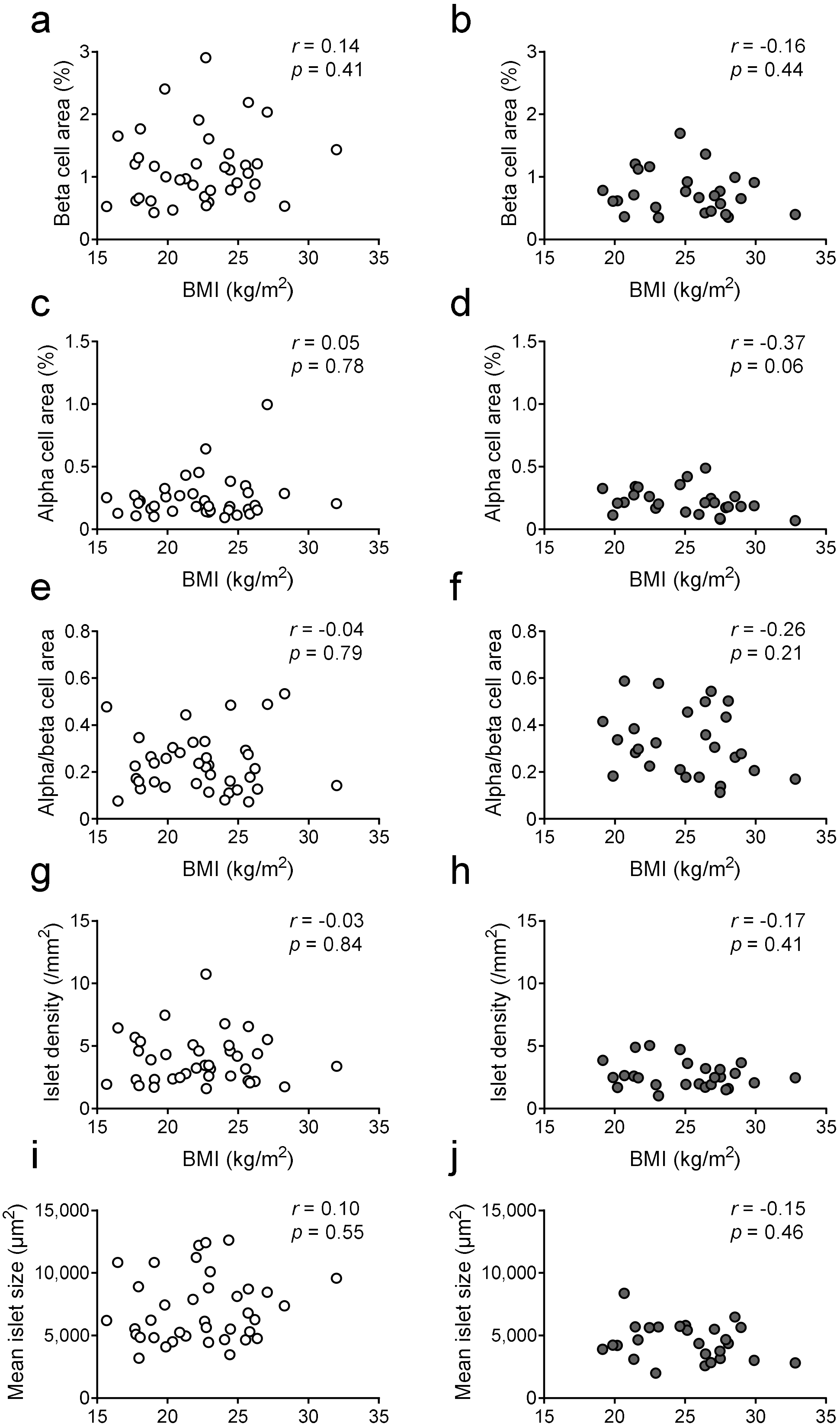

a
r = 0.14
p = 0.41
Beta cell area (%)
BMI (kg/m2)
b
r = -0.16
p = 0.44
Beta cell area (%)
BMI (kg/m2)
c
r = 0.05
p = 0.78
Alpha cell area (%)
BMI (kg/m2)
d
r = -0.37
p = 0.06
Alpha cell area (%)
BMI (kg/m2)
e
r = -0.04
p = 0.79
Alpha/beta cell area
BMI (kg/m2)
f
r = -0.26
p = 0.21
Alpha/beta cell area
BMI (kg/m2)
g
r = -0.03
p = 0.84
Islet density (/mm2)
BMI (kg/m2)
h
r = -0.17
p = 0.41
Islet density (/mm2)
BMI (kg/m2)
i
r = 0.10
p = 0.55
Mean islet size (μm2)
BMI (kg/m2)
j
r = -0.15
p = 0.46
Mean islet size (μm2)
BMI (kg/m2)

ESM Figure 3.

Correlation between current BMI and beta cell area (BCA, a and b), alpha cell area (ACA, c and d), ACA to BCA ratio (e and f), islet density (g and h) and mean islet size (i and j) in participants with (DM group) and without (NDM group) diabetes. Grey and white circles show DM and NDM participants, respectively.

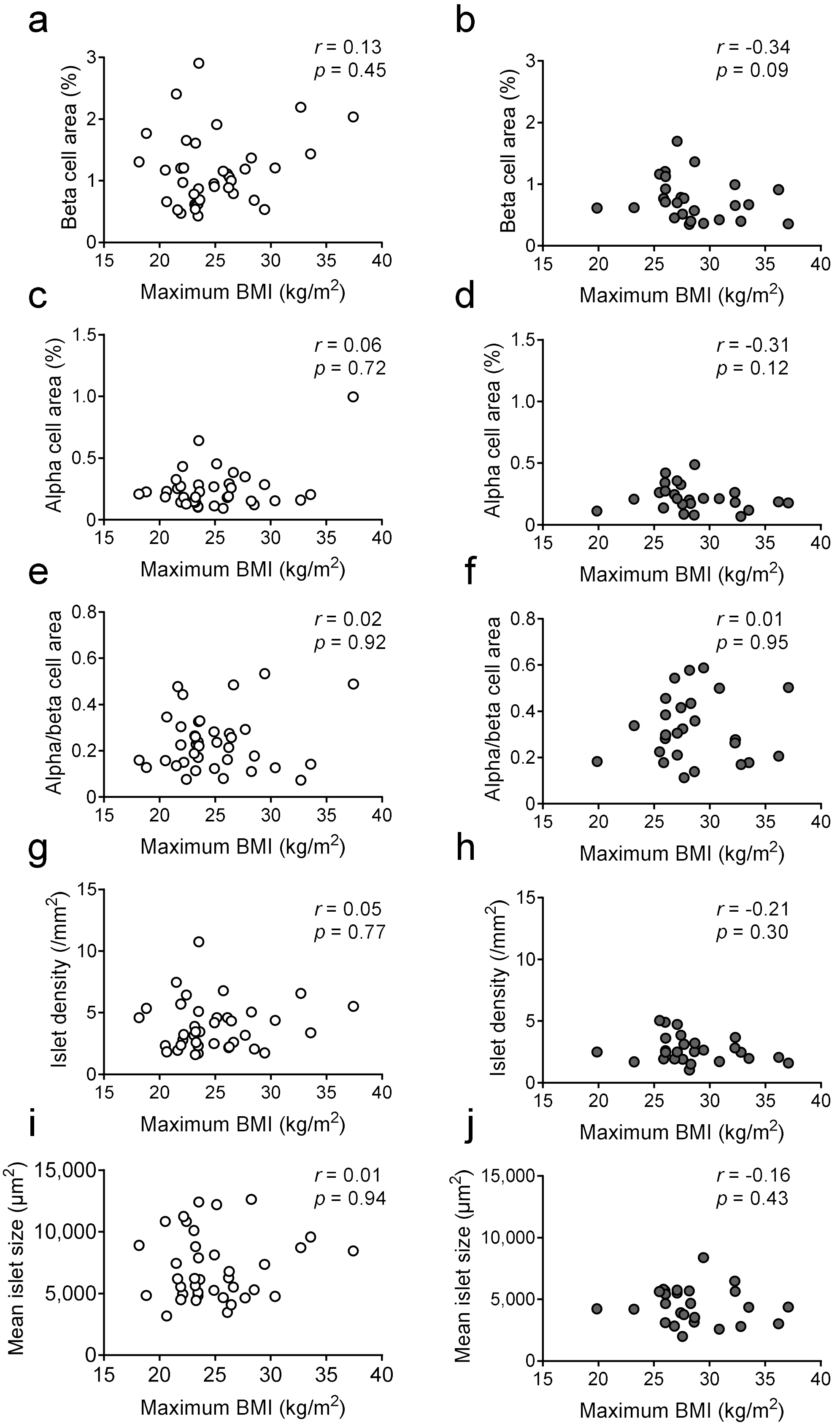

a
r = 0.13
p = 0.45
Beta cell area (%)
Maximum BMI (kg/m2)
b
r = -0.34
p = 0.09
Beta cell area (%)
Maximum BMI (kg/m2)
c
r = 0.06
p = 0.72
Alpha cell area (%)
Maximum BMI (kg/m2)
d
r = -0.31
p = 0.12
Alpha cell area (%)
Maximum BMI (kg/m2)
e
r = 0.02
p = 0.92
Alpha/beta cell area
Maximum BMI (kg/m2)
f
r = 0.01
p = 0.95
Alpha/beta cell area
Maximum BMI (kg/m2)
g
r = 0.05
p = 0.77
Islet density (/mm2)
Maximum BMI (kg/m2)
h
r = -0.21
p = 0.30
Islet density (/mm2)
Maximum BMI (kg/m2)
i
r = 0.01
p = 0.94
Mean islet size (μm2)
Maximum BMI (kg/m2)
j
r = -0.16
p = 0.43
Mean islet size (μm2)
Maximum BMI (kg/m2)

ESM Figure 4.

Correlation between maximum BMI and beta cell area (BCA, a and b), alpha cell area (ACA, c and d), ACA to BCA ratio (e and f), islet density (g and h) and mean islet size (i and j) in participants with (DM group) and without (NDM group) diabetes. Grey and white circles show DM and NDM participants, respectively. Maximum BMI was calculated from maximum body weight in life.

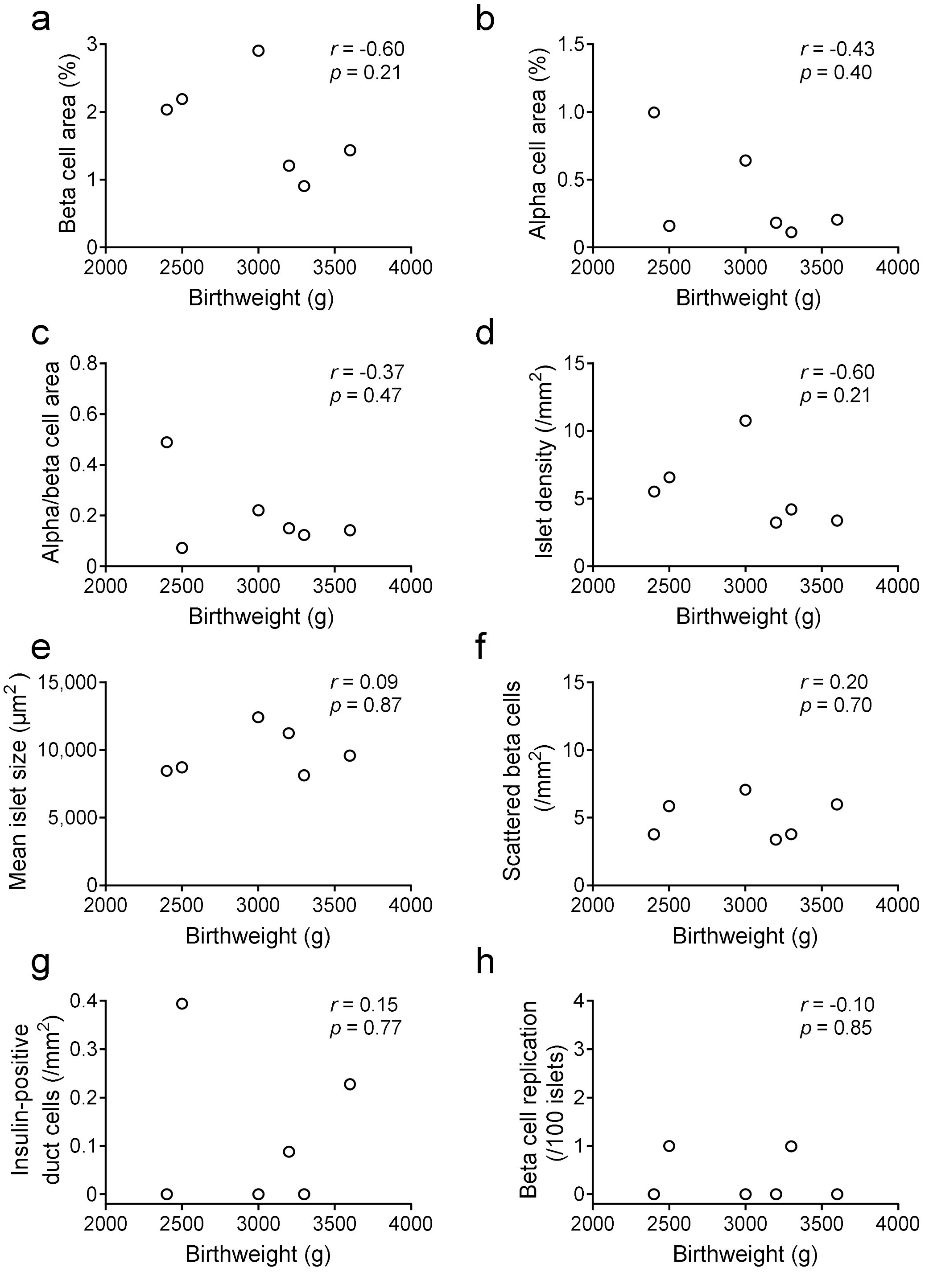

ESM Figure 5.

Correlation between birth weight and beta cell area (BCA, a), alpha cell area (ACA, b), ACA to BCA ratio (c), islet density (d), mean islet size (e) and beta cell turnover (f-h) in non-diabetic participants with history of childhood obesity (NDM-CO group, N = 6).

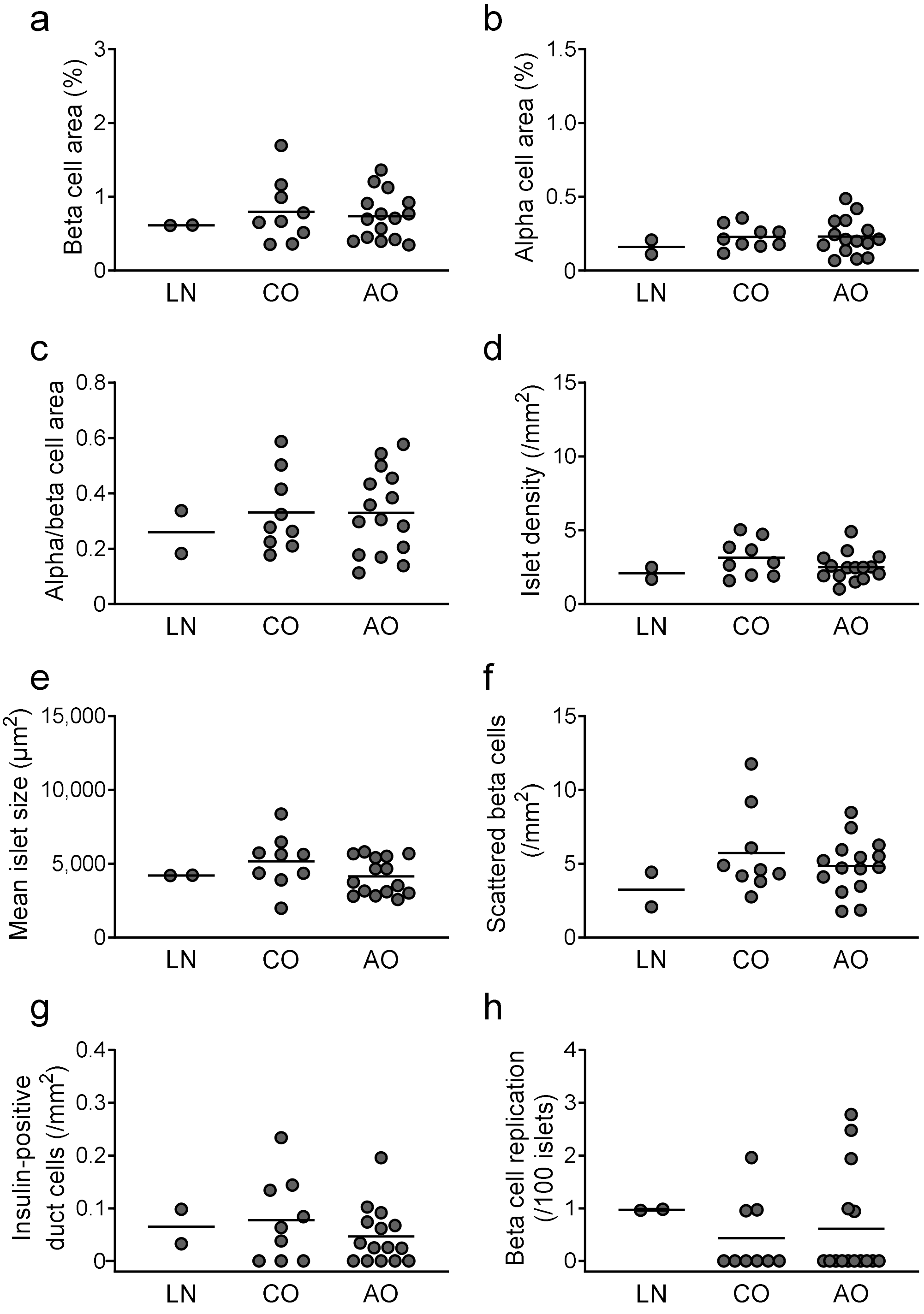

ESM Figure 6.

Effects of history of childhood obesity and adulthood obesity on beta cell area (BCA, a), alpha cell area (ACA, b), ACA to BCA ratio (c), islet density (d), mean islet size (e) and beta cell turnover (f-h) in participants with diabetes (DM group). Bars indicate mean. LN: no history of obesity in childhood or adulthood; CO: history of childhood obesity

regardless of presence or absence of adulthood obesity; AO: no history of childhood obesity but history of adulthood obesity. There were no significant differences between any groups.

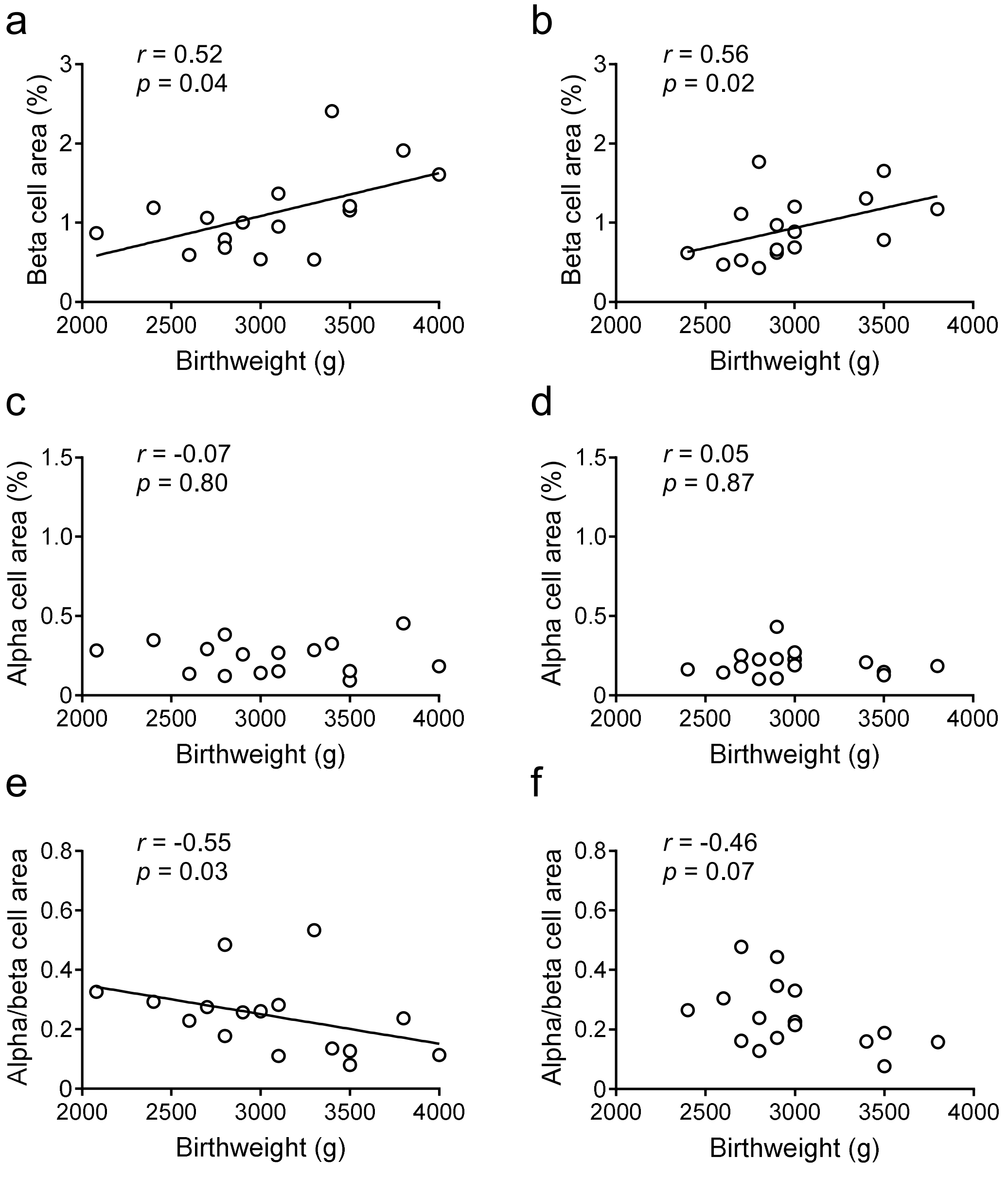

ESM Figure 7.

Correlation between birth weight and beta cell area (BCA), alpha cell area (ACA), and ACA to BCA ratio in non-diabetic participants without history of childhood obesity according to gender (male: a, c and e, female: b, d and f).

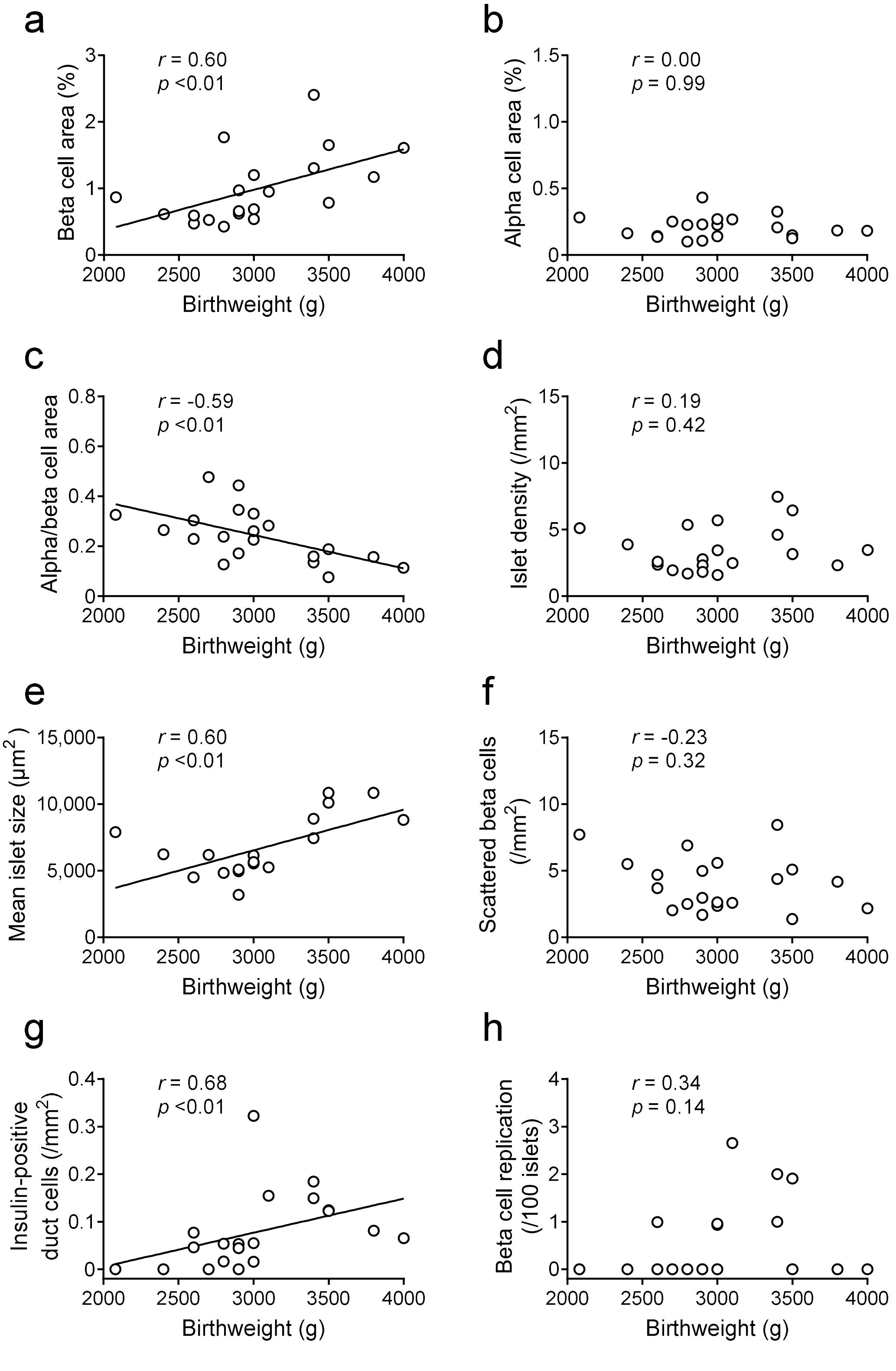

ESM Figure 8.

Correlation between birth weight and beta cell area (BCA, a), alpha cell area (ACA, b),

ACA to BCA ratio (c), islet density (d), mean islet size (e) and beta cell turnover (f-h) in non-diabetic participants without history of obesity (NDM-LN group, N = 20).

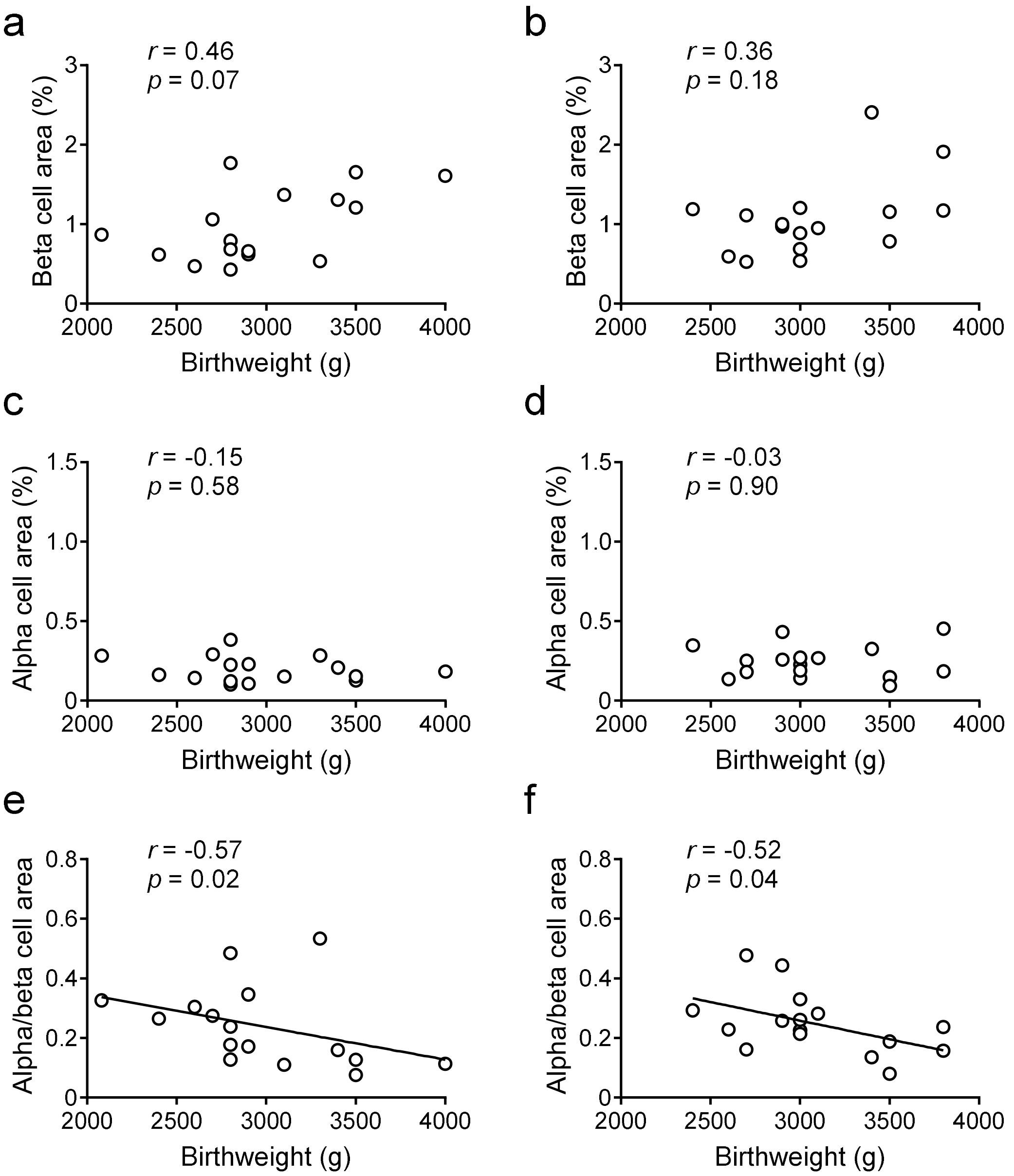

ESM Figure 9.

Correlation between birth weight and beta cell area (BCA), alpha cell area (ACA), and ACA to BCA ratio in non-diabetic participants without history of childhood obesity according to pancreas samples (pancreas head: a, c and e, body/tail: b, d and f).

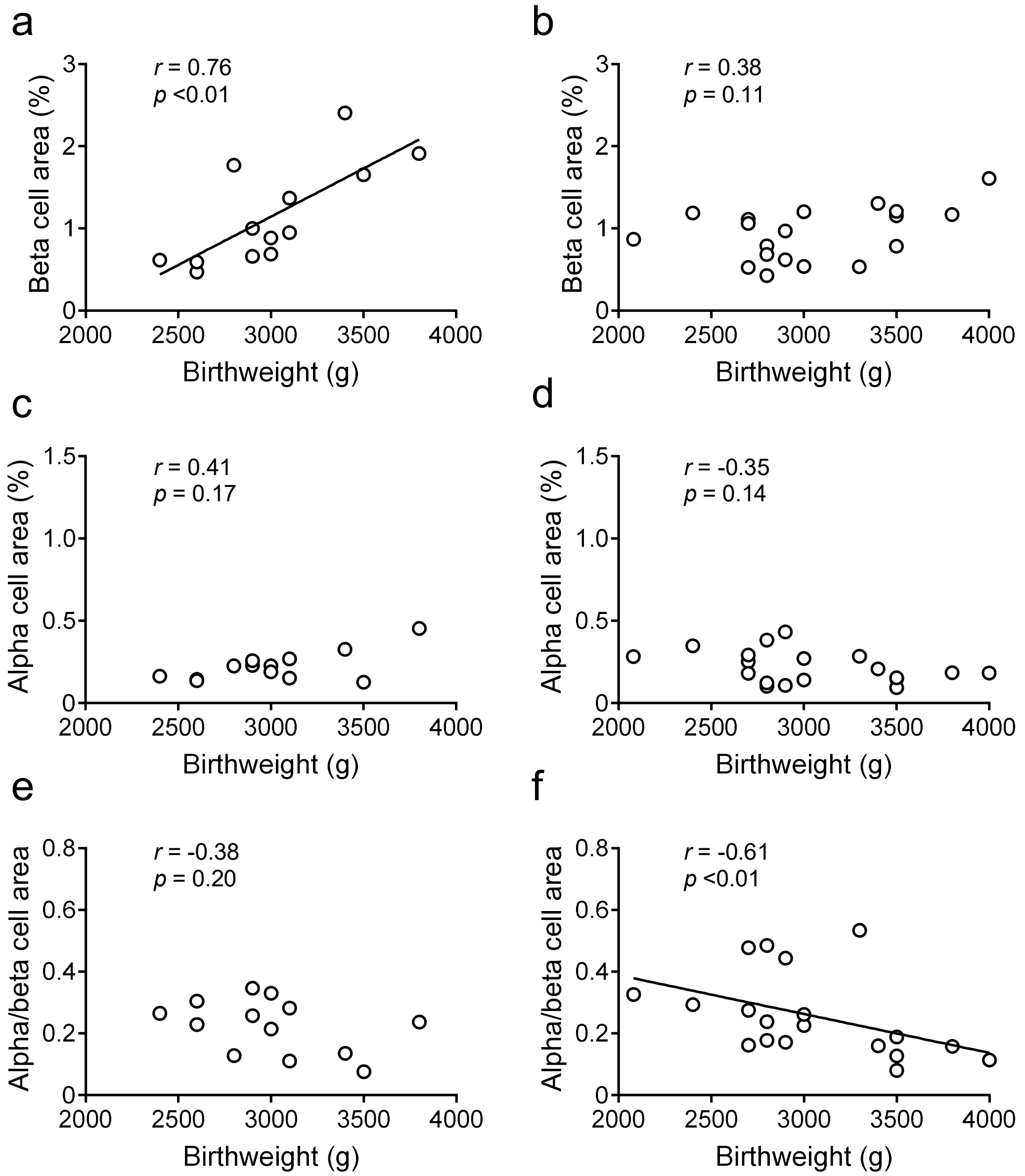

ESM Figure 10.

Correlation between birth weight and beta cell area (BCA), alpha cell area (ACA), and ACA to BCA ratio in non-diabetic participants without history of childhood obesity according to pancreatic diseases (pancreatic cancer: a, c and e, non-pancreatic cancer: b, d and f).
